# Supplementary material for: DNA methylation-based epigenetic signatures predict somatic genomic alterations in gliomas
Source: Nat Commun. 2022 Jul 29;13:4410. doi: 10.1038/s41467-022-31827-x (PMC9338285; doi:10.1038/s41467-022-31827-x)
Supplement: Supplementary file 1 — Supplementary Information [file 41467_2022_31827_MOESM1_ESM.pdf]

Supplementary Figure 1. Investigation of HM450K Probes located on *ATRX*.

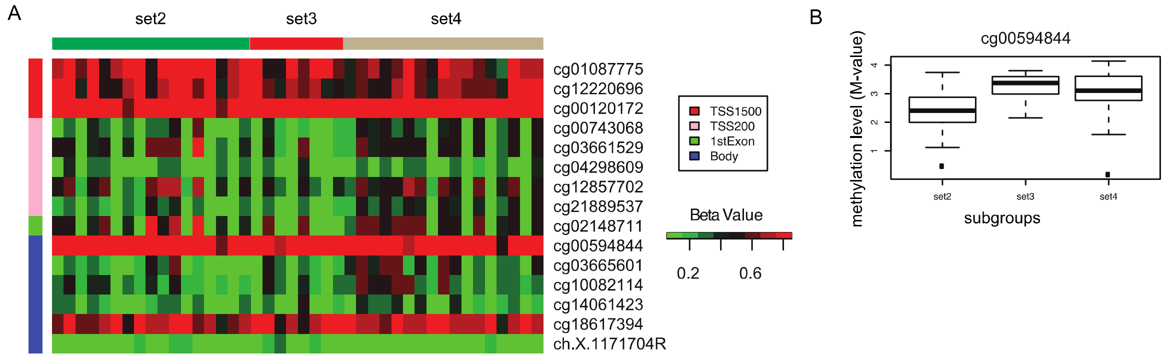

a Heatmap shows the methylation beta value of HM450K probes located at the *ATRX* gene region (TSS200, TSS1500, 1st Exon, and body) for samples in set2, set3, and set4. Each column represents one biologically independent sample (set2: n=17 samples, set3: n=8 samples, set4: n=17 samples) while each row represents one probe. The column-sidebar indicates the subset samples belong to while the row-sidebar indicates the annotations for probes. **b** Boxplot shows the comparison of DNA methylation level of probe cg00594844 among the set2, set3, and set4. Y-axis shows the DNA methylation level (M-value) and X-axis shows the three sets. Box plot center line represents median value, lower and upper hinges represent 25th and 75th percentiles, and lower and upper whiskers represent 1.5 interquartile ranges above and below box limits or maximum/minimum, whichever is closest to median. This is the only probe showed different methylation level (P=0.0213, ANOVA test) among the three subsets.

Supplementary Figure 2. Sample annotation by methyl-based predicted genomic alterations.

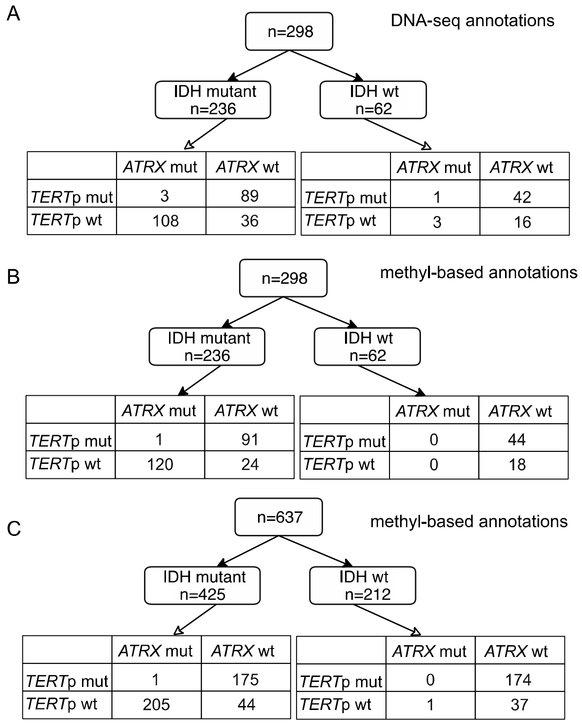

A Samples (n=298) with DNA-seq *IDH* and *ATRX* mutation status and PCR-seq *TERT*p mutation status were split by mutation status. **B** The same 298 samples used in A were split based on the methyl-predicted mutation status of *IDH*, *ATRX*, and *TERT*p. **C** All samples (n=637) used to build *ATRX* prediction models were split based on the methyl-predicted mutation status of *IDH*, *ATRX*, and *TERT*p.

Supplementary Figure 3. CNV profiles derived from HM450K data of samples from the NOA-04 data set.

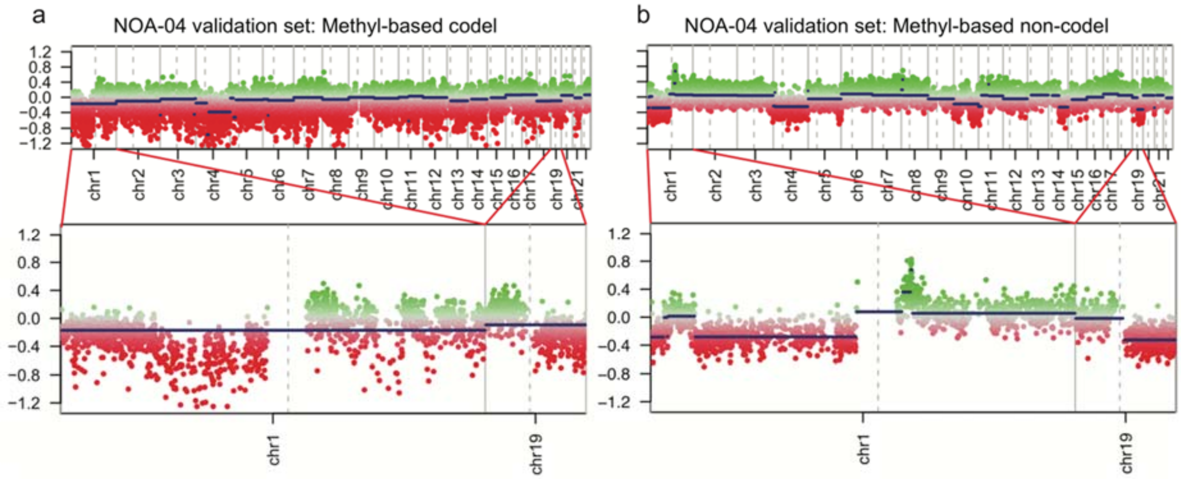

a One NOA-04 sample predicted as 1p19q codel by methyl-based predictive model. **b** One NOA-04 sample predicted as 1p19q non-codel by methyl-based predictive model. We can see this sample has shown only partial deletion in chr1p region.

Supplementary Figure 4. Data simulation procedures for evaluating HM450K data normalization methods

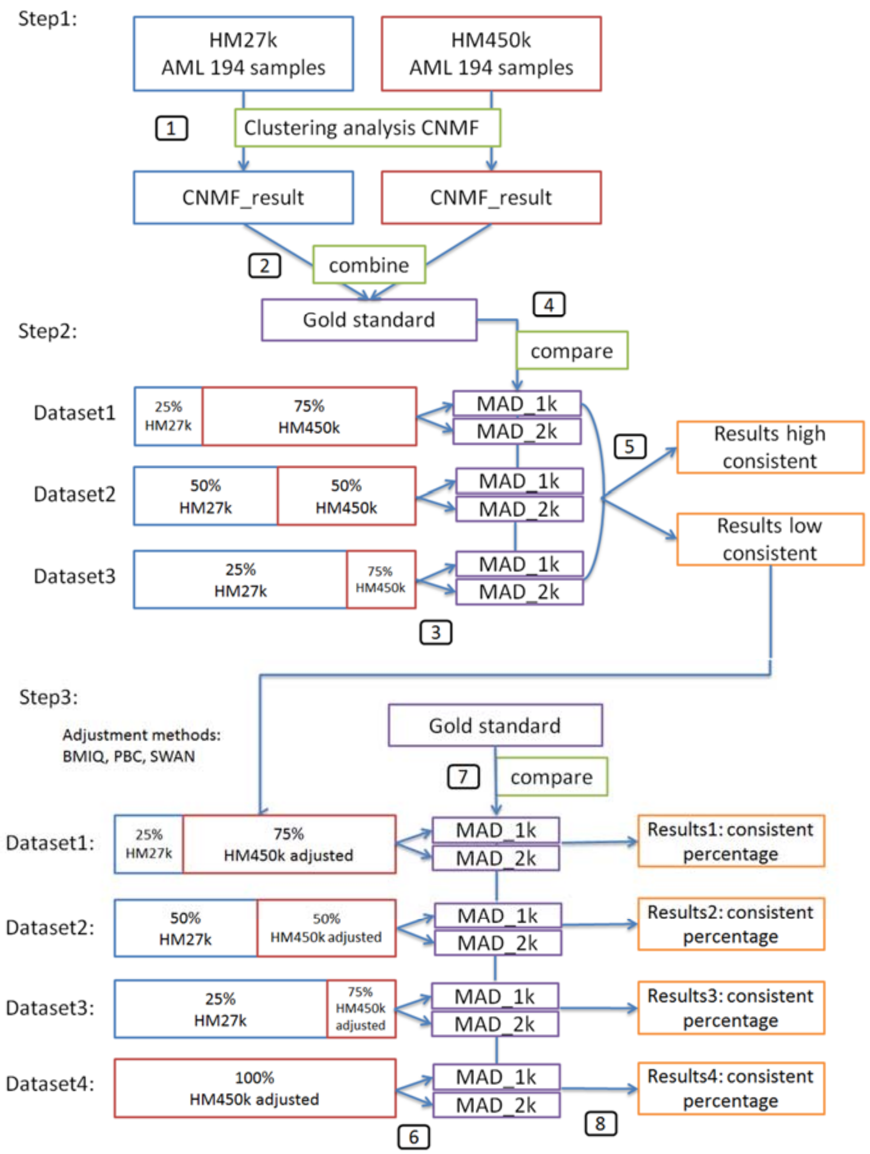

Step 1: Generate the gold standard. Step 2: Evaluate simulated datasets with unadjusted HM450K data. Step 3: Evaluate simulated datasets with adjusted HM450K data. Adjustment methods include BMIQ, PBC, and SWAN. **1**: Use data from HM27K or HM450K to do the clustering analysis with CNMF, obtain the membership for each platform. **2**: Combine the membership results from each dataset and generate the final results as the gold standard. **3**: Simulate three datasets with HM27K and unadjusted HM450K data, create two subsets for each dataset, and obtain the clustering results for each subset. **4**: Compare the six results with gold standard. **5**: Obtain the concordance percentage. If the concordance percentage is high, the data from HM450K will be used without any adjustment. If the concordance percentage is low, three published adjustment methods will be evaluated (BMIQ, PBC, and SWAN). **6**: For each adjustment method, four datasets are simulated. Two subsets are generated by MAD value. CNMF is applied for each subset. **7**: Compare the membership results with the gold standard. **8**: Generate the consistent percentage for each simulated dataset.

Supplementary Figure 5. Prediction models' performance of IDH mutation in training set.

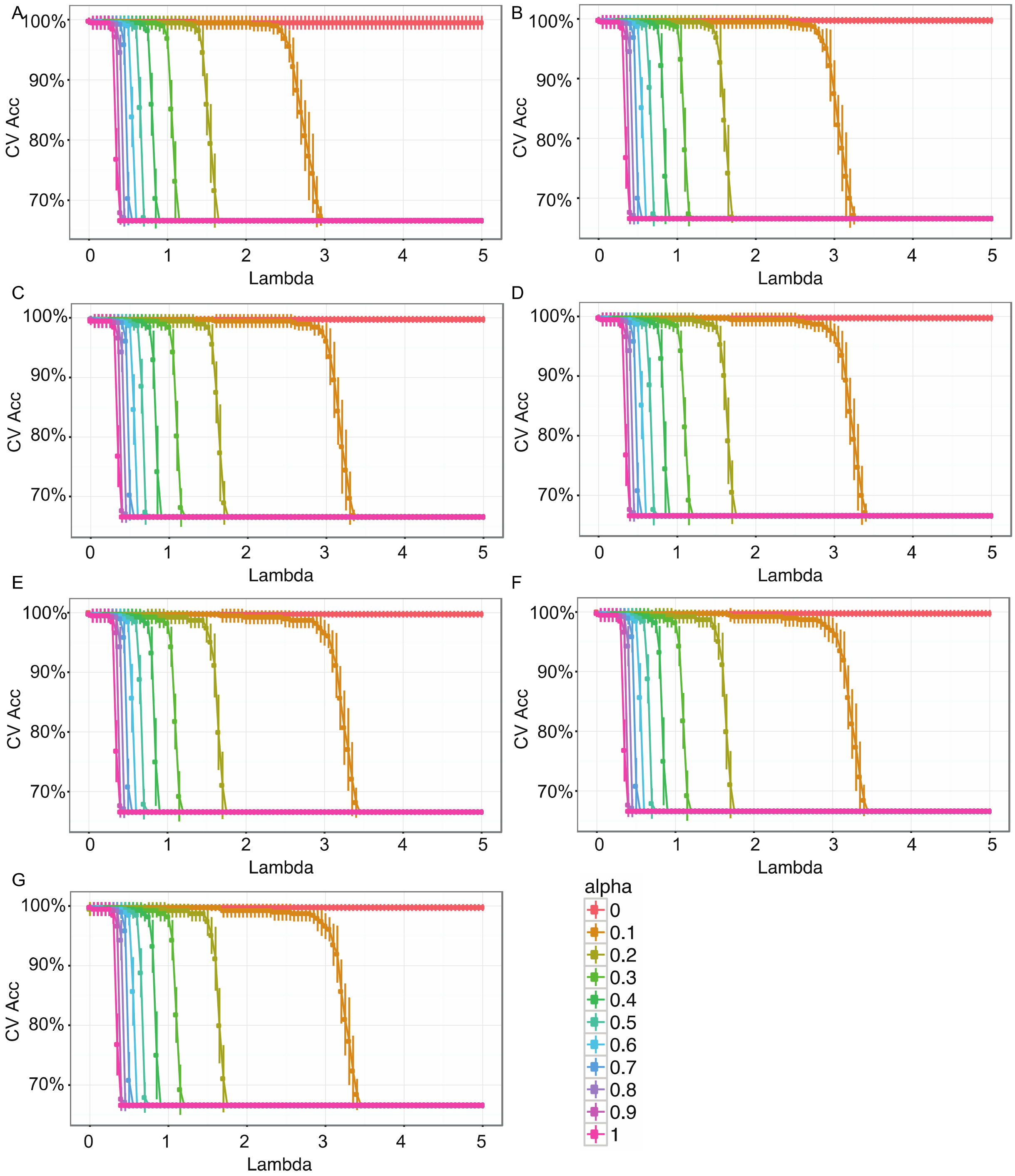

Each figure used a different number of probes and different alpha and lambda combinations. The x-axis represents the lambda value and y-axis represent the prediction accuracy among the 5-fold CV. Lines with different colors represent the different alpha value as shown in the legend. Figure **A** to **G** show the prediction accuracy using the top 20, 50, 100, 200, 500, 1000, and 1500 probes when fitting the model, respectively.

Supplementary Figure 6. Prediction models' performance of *TERT*p mutation in training set.

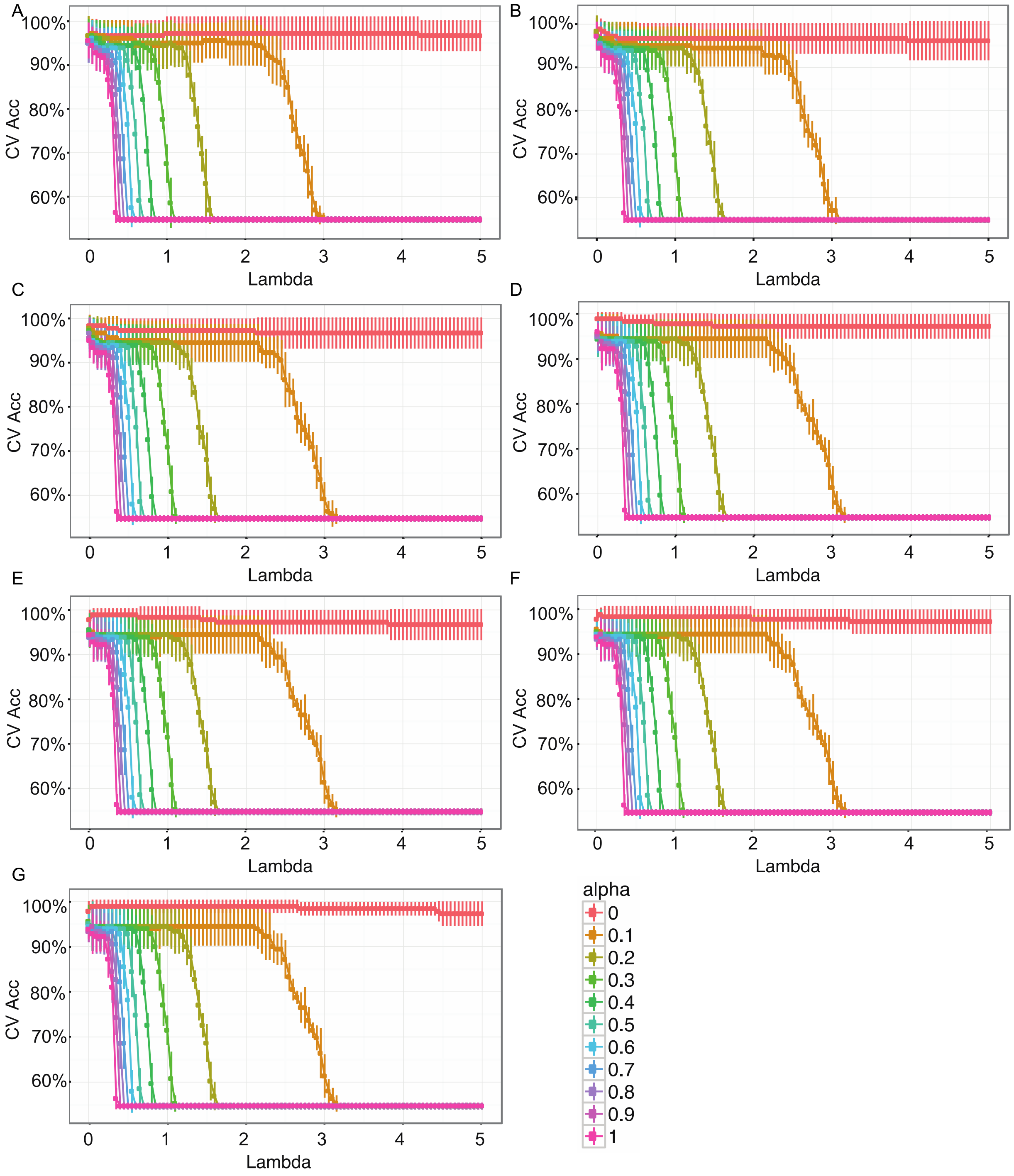

Each figure used different number of probes and different alpha and lambda combinations. The x-axis represents the lambda value and y-axis represent the prediction accuracy among the 5-fold CV. Lines with different colors represent the different alpha value as shown in the legend. Figure **A** to **G** show the prediction accuracy using the top 50, 100, 200, 500, 1000, 1500, and 2000 probes when fitting the model, respectively.

Supplementary Figure 7. Prediction models' performance of *ATRX* mutation in training set.

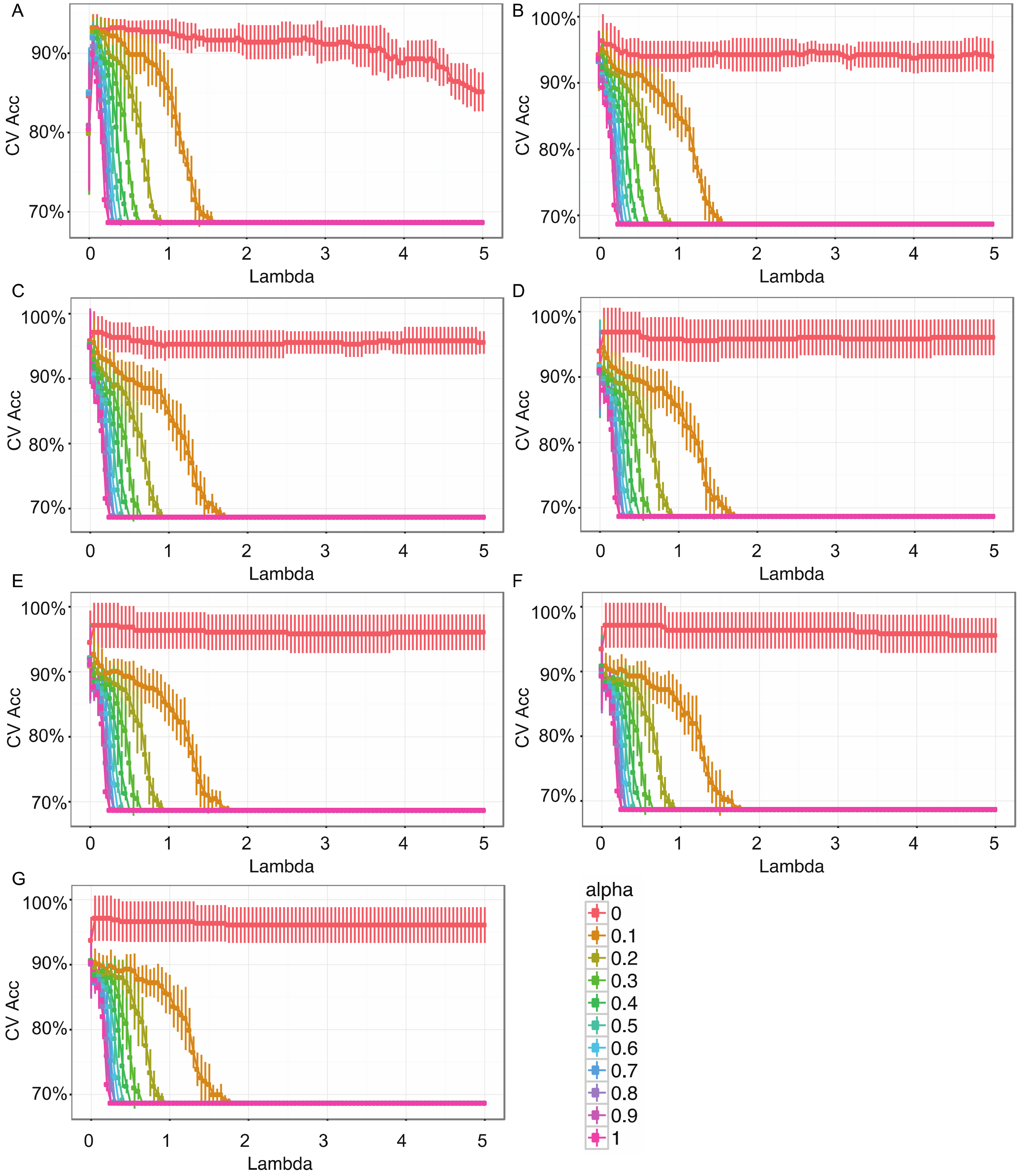

Each figure used different number of probes and different alpha and lambda combinations. The x-axis represents the lambda value and y-axis represent the prediction accuracy among the 5-fold CV. Lines with different colors represent the different alpha value as shown in the legend. **A** to **G** shown the prediction accuracy using the top 50, 100, 200, 500, 1000, 1500, and 2000 probes when fitting the model, respectively.

Supplementary Figure 8. Prediction models' performance of Chromosome 1p/19q co-deletion in training set.

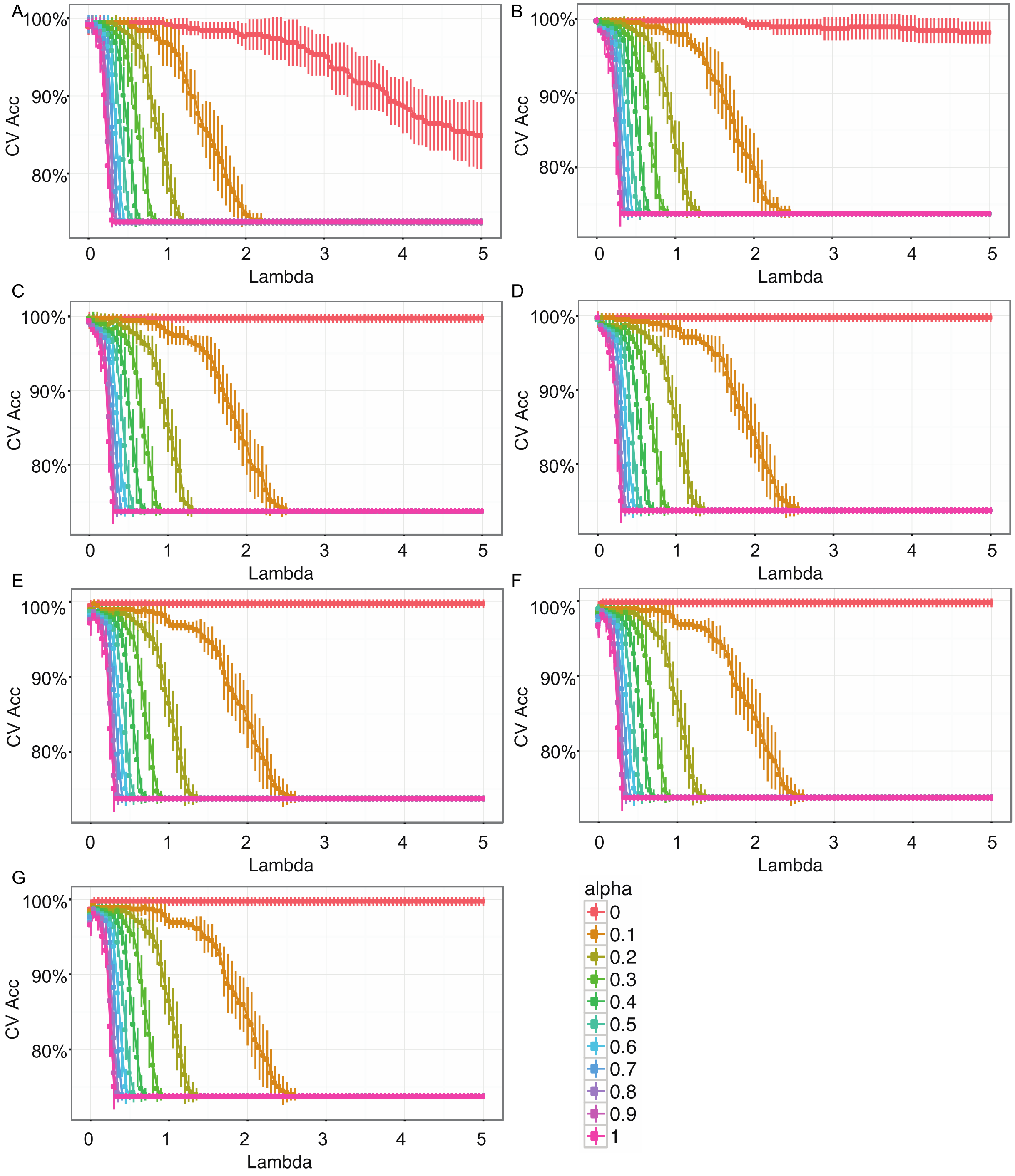

Each figure used different number of probes and different alpha and lambda combinations. The x-axis represents the lambda value and y-axis represent the prediction accuracy among the 5-fold CV. Lines with different colors represent the different alpha value as shown in the legend. **A** to **G** show the prediction accuracy using the top 20, 50, 100, 200, 500, 1000, and 1279 probes when fitting the model, respectively.

Supplementary Figure 9. Comparison of misclassified rate of TCGA gene expression subtype of 5-fold cross validation tests using top quantile probes.

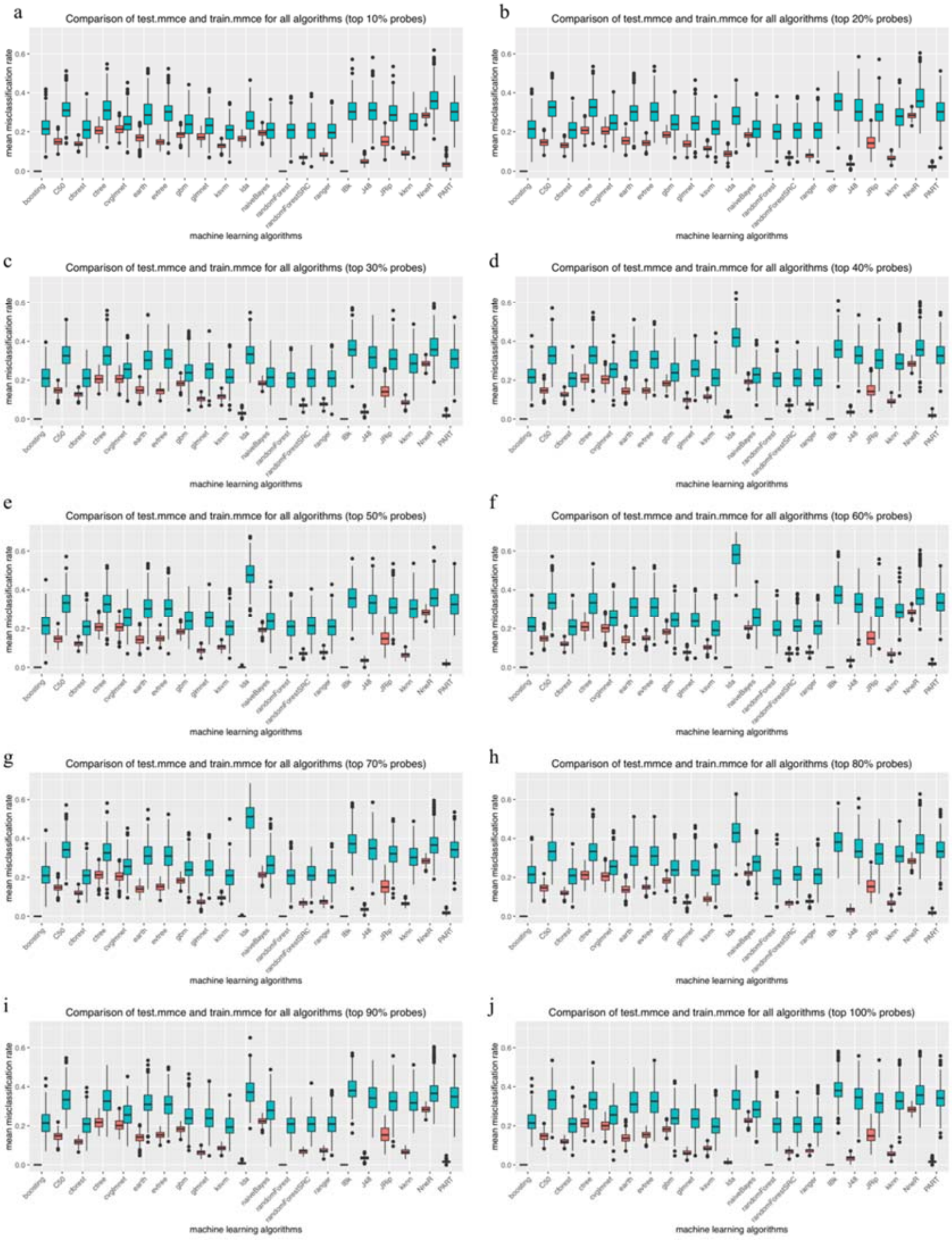

For each machine learning algorithm shown in the x-axis, two boxplots were shown: the red boxplot shows the misclassification rate when building the model with randomly chosen four folds of the training data set and the green boxplot shows the misclassication rate when applying the model to the remaining one fold of the training data set. Y-axis is the averaged misclassification rate among 5-fold CV. Figures **a** to **j** represent the top 10%, 20%, 30%, 40%, 50%, 60%, 70%, 80%, 90%, and 100% probes, respectively. Each bar represents a summary of n=500 data points generated from 5-fold CV performed 100 times. Box plot center line represents median value, lower and upper hinges represent 25th and 75th percentiles, and lower and upper whiskers represent 1.5 interquartile ranges above and below box limits or maximum/minimum, whichever is closest to median.

Supplementary Figure 10. Summarization of the averaged misclassification rate for the 5-fold CV using top quantile probes.

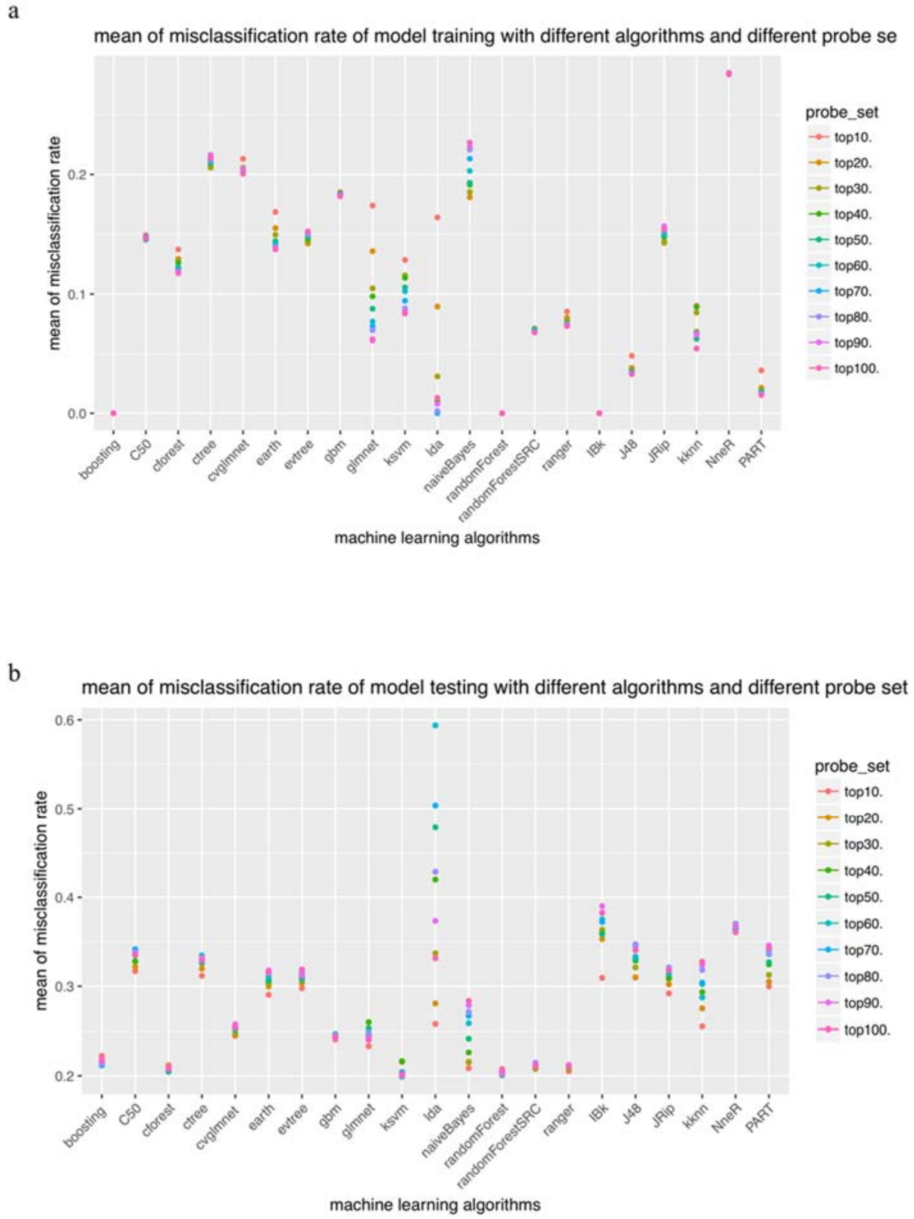

The x-axis shows the twenty-one machine learning algorithms, and the y-axis shows the averaged misclassification rate. The different colored dots represent the summarized the mean of the misclassification rate calculated with different probe sets. **a.** The averaged misclassification rate of the model building using random four folds of data in the training set. **b.** The averaged misclassification rate when applying the model to predict the remaining one fold of data in the training set. The means represent averages of n=500 data points, as in Supplementary Figure 5.

Supplementary Figure 11. The summarized sum of probability deviations among twenty-one machine learning algorithms using different top quantile probe sets.

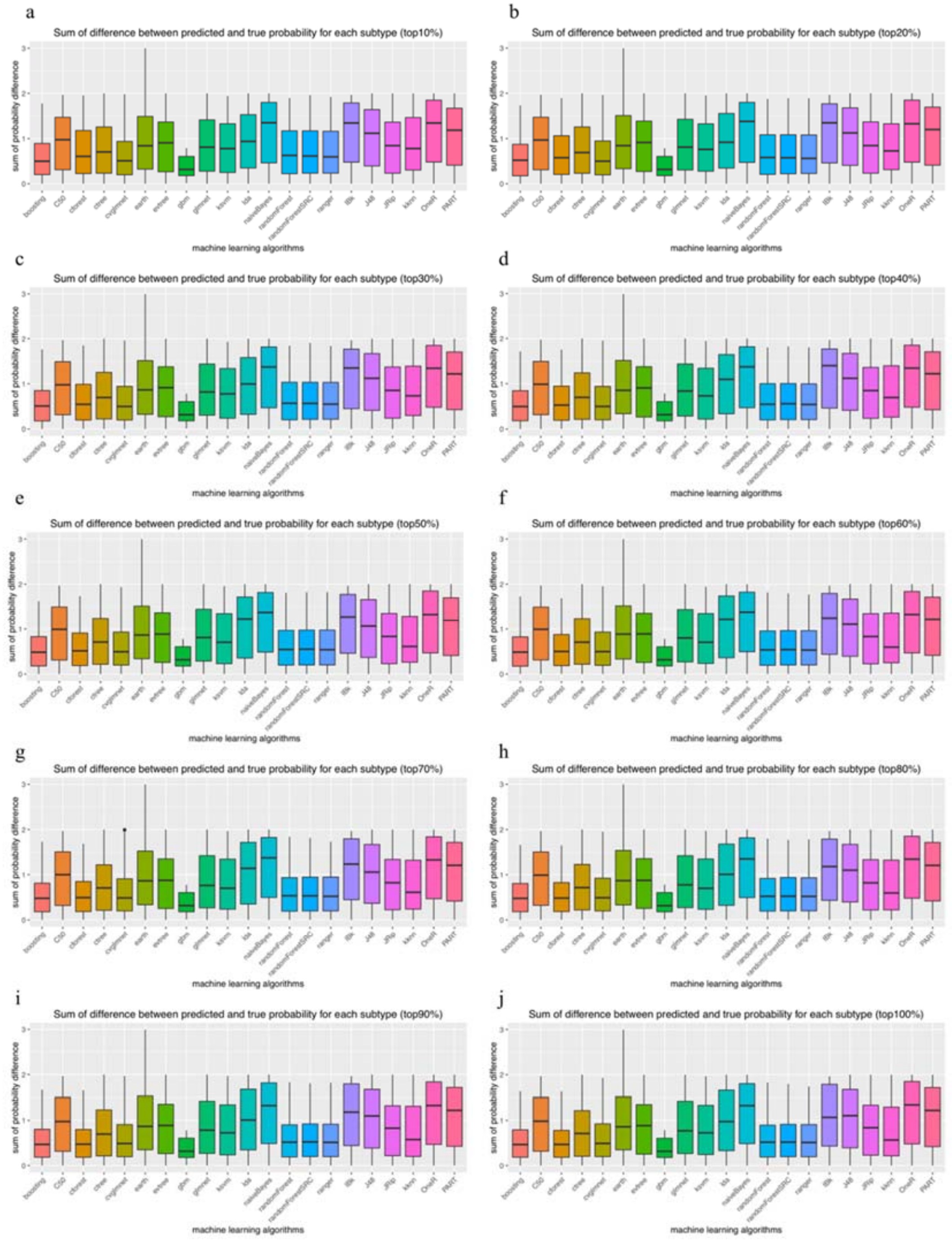

The x-axis shows the evaluated algorithms and y-axis shows the sum of probability deviations. Each boxplot represent the summary of the sum of probability deviations in the test-fold among the 5-fold CV among hundreds repeated calculations. Figure **a** to **j** show the results of top 10%, 20%, 30%, 40%, 50%, 60%, 70%, 80%, 90%, and 100% quantile probe sets, respectively. Each bar represents n=21,200 data points, for 100 attempts at classification of each of 212 samples. Box plot center line represents median value, lower and upper hinges represent 25th and 75th percentiles, and lower and upper whiskers represent 1.5 interquartile ranges above and below box limits or maximum/minimum, whichever is closest to median.

Supplementary Figure 12. Summarized averaged sum of probability deviations among 5-fold CV using different top quantile probe sets.

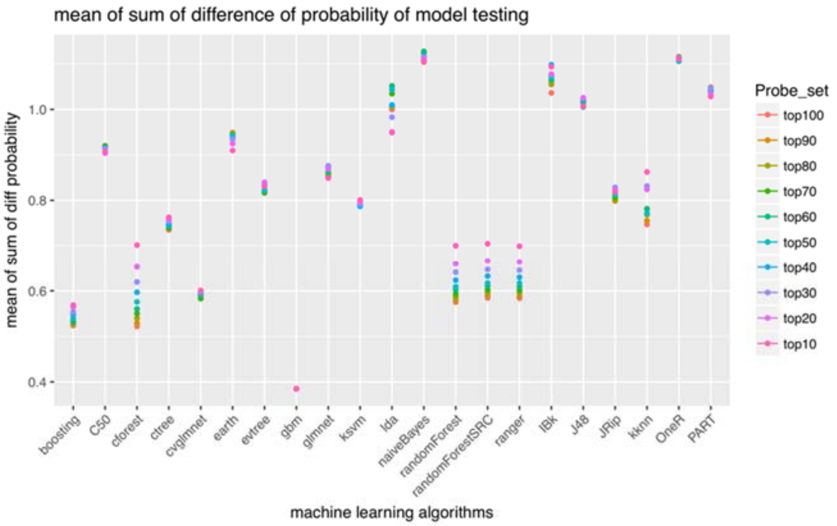

X-axis represents the twenty-one machine learning algorithms and y-axis represents the averaged sum of probability deviations. Each colored dot represents the averaged sum of probability deviations (for n = 21,200 data points, as in Supplementary Figure 7) using different probe sets.

Supplementary Figure 13. Tumor purity comparison between training, development, and tests set for each biomarker.

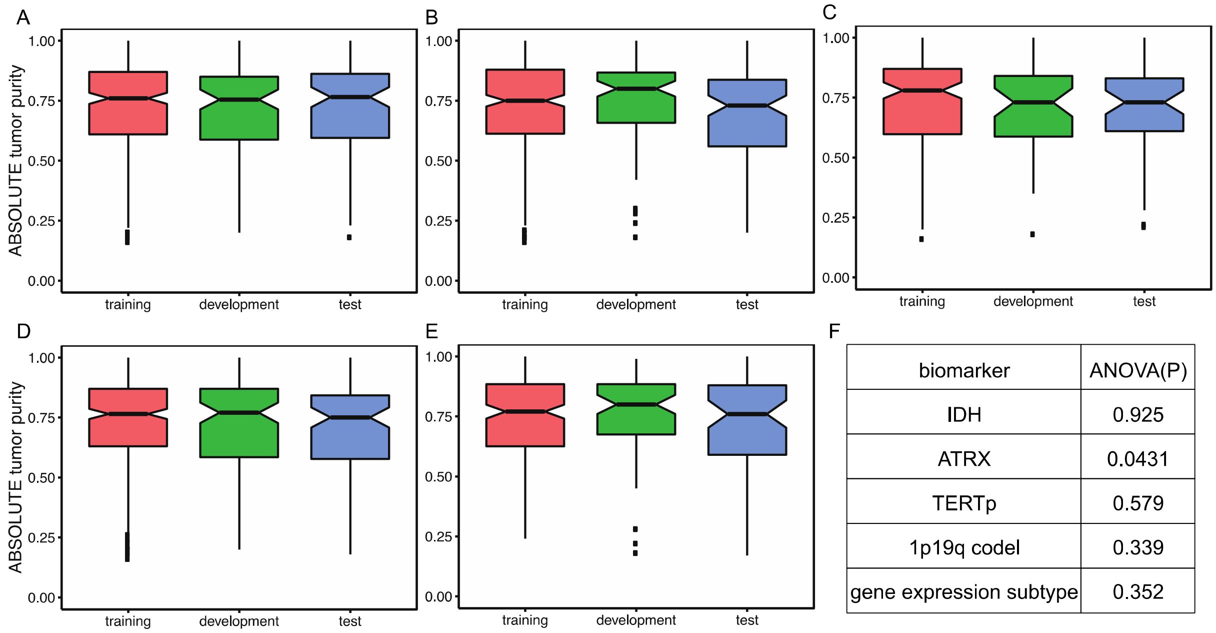

F

biomarker	ANOVA(P)
IDH	0.925
ATRX	0.0431
TERTp	0.579
1p19q codel	0.339
gene expression subtype	0.352

From A to E, each Figure shows the tumor purity compared between training, development, and test sets for samples used in *IDH*, *ATRX*, *TERT*p, 1p19q codel, and gene expression subtypes predictive model development. Y-axis is the tumor purity obtained from ABSOLUTE using whole-exome sequencing data. X-axis is the three subsets data used in model development. The number of samples included in each plot are: (A) n=383 training, n=127 development, n=127 test samples. (B) n=383 training, n=126 development, n=126 test samples. (C) n=179 training, n=60 development, n=59 test samples. (D) n=385 training, n=129 development, n=127 test samples. (E) n=212 training, n=72 development, n=72 test samples. Box plot center line represents median value, lower and upper hinges represent 25th and 75th percentiles, and lower and upper whiskers represent 1.5 interquartile ranges above and below box limits or maximum/minimum, whichever is closest to median. Figure F shows the P-value of a one-way ANOVA test comparing the tumor purity between the three subsets.

Supplementary Table 1. CpG island relationship enrichment for prediction signatures including binary genomic alterations and gene expression subtypes

Genomic alteration	Relation_to_CpG_island	# probes available	# probes	Normalization to total #probes	Percentage (sum to 1)	Two-sided p-value (proportion test)
IDH	Island	120312	89	0.074%	75.9958%	1.431e-31
	N_Shelf	18946	0	0.000%	0.0000%	
	N_Shore	50338	5	0.010%	10.2043%	
	S_Shelf	16765	0	0.000%	0.0000%	
	S_Shore	39412	5	0.013%	13.0332%	
	not categorized	134237	1	0.001%	0.7653%	

Genomic alteration	Relation_to_CpG_island	# probes available	# probes	Normalization to total #probes	Percentage which sum to 1	Two-sided p-value (proportion test)
TERTp	Island	120312	536	0.446%	33.104%	1.531e-46
	N_Shelf	18946	30	0.158%	11.766%	
	N_Shore	50338	100	0.199%	14.762%	
	S_Shelf	16765	31	0.185%	13.740%	
	S_Shore	39412	74	0.188%	13.952%	
	not categorized	134237	229	0.171%	12.676%	

Genomic alterations	Relation_to_CpG_island	# probes available	# probes	Normalization to total #probes	Percentage which sum to 1	Two-sided p-value (proportion test)
ATRX	Island	120312	261	0.217%	31.313%	2.201e-24
	N_Shelf	18946	18	0.095%	13.713%	
	N_Shore	50338	71	0.141%	20.359%	
	S_Shelf	16765	6	0.036%	5.166%	
	S_Shore	39412	54	0.137%	19.777%	
	not categorized	134237	90	0.067%	9.677%	

Genomic alterations	Relation_to_CpG_island	# probes available	# probes	Normalization to total #probes	Percentage which sum to 1	Two-sided p-value (proportion test)
1p19q codel	Island	120312	53	0.044%	32.777%	0.0001692
	N_Shelf	18946	1	0.005%	3.927%	
	N_Shore	50338	14	0.028%	20.693%	
	S_Shelf	16765	4	0.024%	17.752%	
	S_Shore	39412	7	0.018%	13.215%	
	not categorized	134237	21	0.016%	11.640%	

Island: CpG island; Shelf: 2-4kb form island; Shore: 1-2kb from island; N: upstream of CpG island; S: downstream of CpG island.
For Gene expression subtype, because all probes were pre-selected for CpG island, therefore, we are not providing the information here.

Supplementary Table 2. Gene structure enrichment for prediction signatures including binary genomic alterations and gene expression subtypes

Genomic Alterations	relation to gene structure	# probes available	#probes	normalized #probe	Percentage (sum to 1)	Two-sided p-value (proportion test)
	TSS200	41774	20	0.048%	22.055%	
	TSS1500	55088	13	0.024%	10.871%	
	Body	126827	23	0.018%	8.354%	
IDH	3'UTR	13641	0	0.000%	0.000%	0.00004473
	5'UTR	33719	14	0.042%	19.127%	
	1st Exon	18147	12	0.066%	30.462%	
	not categorized	90814	18	0.020%	9.131%	

Genomic Alterations	relation to gene structure	# probes available	#probes	normalized #probe	Percentage (sum to 1)	Two-sided p-value (proportion test)
	TSS200	41774	162	0.388%	16.700%	
	TSS1500	55088	130	0.236%	10.162%	
	Body	126827	291	0.229%	9.881%	
TERTp	3'UTR	13641	96	0.704%	30.306%	1.527e-38
	5'UTR	33719	34	0.101%	4.342%	
	1st Exon	18147	79	0.435%	18.747%	
	not categorized	90814	208	0.229%	9.863%	

Genomic Alterations	relation to gene structure	# probes available	#probes	normalized #probe	Percentage (sum to 1)	Two-sided p-value (proportion test)
	TSS200	41774	71	0.170%	14.037%	
	TSS1500	55088	67	0.122%	10.044%	
	Body	126827	139	0.110%	9.051%	
ATRX	3'UTR	13641	55	0.403%	33.299%	1.846e-26
	5'UTR	33719	10	0.030%	2.449%	
	1st Exon	18147	46	0.253%	20.934%	
	not categorized	90814	112	0.123%	10.185%	

Genomic Alterations	relation to gene structure	# probes available	#probes	normalized #probe	Percentage (sum to 1)	Two-sided p-value (proportion test)
	TSS200	41774	16	0.038%	17.652%	
	TSS1500	55088	19	0.034%	15.896%	
	Body	126827	24	0.019%	8.722%	
chr 1p19q	3'UTR	13641	2	0.015%	6.757%	0.005519
	5'UTR	33719	15	0.044%	20.503%	
	1st Exon	18147	9	0.050%	22.858%	
	not categorized	90814	15	0.017%	7.613%	

Genomic Alterations	relation to gene structure	# probes available	#probes	normalized #probe	Percentage (sum to 1)	Two-sided p-value (proportion test)
	TSS200	41774	38	0.091%	11.990%	
	TSS1500	55088	54	0.098%	12.920%	
	Body	126827	62	0.049%	6.443%	
gene expression subtype	3'UTR	13641	3	0.022%	2.899%	2.261e-59
	5'UTR	33719	66	0.196%	25.799%	
	1st Exon	18147	55	0.303%	39.948%	
	not categorized	90814	0	0.000%	0.000%	

TSS: transcriptional start site

Supplementary Table 3. ***ATRX*** **prediction results analysis for misclassified samples**

sample	ATRX status		Find in algorithms (Yes/No)				#detected times	IDH status
	DNA-seq	Methyl	Muse	somatic sniper	Varscan	Mutect		
TCGA-WY-A85C-01A	WT	mutant	No	No	No	No	0	Mutant
TCGA-WY-A859-01A	WT	mutant	No	No	No	No	0	Mutant
TCGA-WH-A86K-01A	WT	mutant	No	No	No	No	0	Mutant
TCGA-S9-A7R4-01A	WT	mutant	No	No	No	No	0	Mutant
TCGA-P5-A5F4-01A	WT	mutant	No	No	No	No	0	Mutant
TCGA-DH-5143-01A	WT	mutant	No	No	No	No	0	Mutant
TCGA-DB-5277-01A	WT	mutant	No	No	No	No	0	Mutant
TCGA-CS-5393-01A	WT	mutant	No	No	No	No	0	Mutant
TCGA-26-1442-01A-01D	WT	mutant	No	Yes	No	No	1	Mutant
TCGA-CS-6665-01A-11D	WT	mutant	No	No	No	Yes	1	Mutant
TCGA-HT-7606-01A-11D	WT	mutant	No	No	No	Yes	1	Mutant
TCGA-HT-8018-01A-11D	WT	mutant	No	No	No	Yes	1	Mutant
TCGA-HW-A5KM-01A	WT	mutant	No	No	No	Yes	1	Mutant
TCGA-S9-A7R3-01A-11D	WT	mutant	No	No	No	Yes	1	Mutant
TCGA-S9-A89Z-01A-11D	WT	mutant	No	No	No	Yes	1	Mutant
TCGA-TQ-A7RF-01A	WT	mutant	No	No	No	Yes	1	Mutant
TCGA-TQ-A7RW-01A	WT	mutant	No	No	No	Yes	1	Mutant
TCGA-TQ-A8XE-01A	WT	mutant	No	No	No	Yes	1	Mutant
TCGA-06-6389-01A-11D	WT	mutant	No	No	Yes	Yes	2	Mutant
TCGA-DB-A4XB-01A	WT	mutant	No	Yes	No	Yes	2	Mutant
TCGA-S9-A7J0-01A-11D	WT	mutant	No	No	Yes	Yes	2	Mutant
TCGA-DU-8167-01A-11D	WT	mutant	Yes	No	Yes	Yes	3	Mutant
TCGA-DB-A4X9-01A-11D	WT	mutant	Yes	Yes	Yes	Yes	4	Mutant
TCGA-HT-7601-01A-11D	WT	mutant	Yes	Yes	Yes	Yes	4	Mutant
TCGA-HW-8321-01A	WT	mutant	Yes	Yes	Yes	Yes	4	Mutant
TCGA-06-A5U0-01A-11D	mutant	WT	No	No	Yes	Yes	2	WT
TCGA-DU-7298-01A-11D	mutant	WT	No	No	Yes	Yes	2	Mutant
TCGA-HT-7469-01A-11D	mutant	WT	No	No	Yes	Yes	2	WT
TCGA-HT-7857-01A-11D	mutant	WT	No	No	Yes	Yes	2	WT
TCGA-DU-5852-01A-11D	mutant	WT	No	Yes	Yes	Yes	3	WT
TCGA-S9-A89V-01A-11D	mutant	WT	Yes	No	Yes	Yes	3	WT
TCGA-06-5858-01A-01D	mutant	WT	Yes	Yes	Yes	Yes	4	WT
TCGA-06-6388-01A-12D	mutant	WT	Yes	Yes	Yes	Yes	4	WT
TCGA-06-6391-01A-11D	mutant	WT	Yes	Yes	Yes	Yes	4	WT
TCGA-4W-AA9T-01A-11D	mutant	WT	Yes	Yes	Yes	Yes	4	WT
TCGA-74-6575-01A-11D	mutant	WT	Yes	Yes	Yes	Yes	4	WT
TCGA-DH-5144-01A-01D	mutant	WT	Yes	Yes	Yes	Yes	4	Mutant
TCGA-DU-6392-01A-11D	mutant	WT	Yes	Yes	Yes	Yes	4	WT
TCGA-FG-5963-01A-11D	mutant	WT	Yes	Yes	Yes	Yes	4	WT
TCGA-FG-7638-01B-11D	mutant	WT	Yes	Yes	Yes	Yes	4	Mutant
TCGA-FG-A713-01A-11D	mutant	WT	Yes	Yes	Yes	Yes	4	Mutant
TCGA-HT-7880-01A-11D	mutant	WT	Yes	Yes	Yes	Yes	4	Mutant

Supplementary Table 4. MGMT promoter methylation status comparison between Methylation-specific PCR and MGMT-STP27 in NOA04 samples

MGMT-STP27 results	Methylation-specific PCR results	
	Methylated	Unmethylated
Unmethylated	3	13
Methylated	69	29

Supplementary Table 5. Clinical characteristics of cases used for predictive model building.

		LGG	GBM
**n**		516	129
**Sex**	Male	285 (55.2%)	73 (56.6%)
	Female	230 (44.6%)	54 (41.9%)
	Unknown	1 (0.2%)	2 (1.6%)
**Age**	< 18	2 (0.4%)	0 (0%)
	18-29	77 (14.9%)	3 (2.3%)
	30-49	271 (52.5%)	19 (14.7%)
	50-69	148 (28.7%)	71 (55.0%)
	70+	16 (3.1%)	34 (26.4%)
	Unknown	2 (0.4%)	2 (1.6%)
**Race**	White	475 (92.1%)	97 (75.2%)
	Black / African American	21 (4.1%)	23 (17.8%)
	Asian	8 (1.6%)	0 (0%)
	American Indian / Alaska Native	1 (0.2%)	0 (0%)
	Unknown	11 (2.1%)	9 (7.0%)
**Ethnicity**	Not Hispanic or Latino	449 (87.0%)	86 (66.7%)
	Hispanic or Latino	32 (6.2%)	1 (0.8%)
	Unknown	15 (2.9%)	42 (32.6%)
**Grade**	2	249 (48.3%)	-
	3	265 (51.4%)	
	Unknown	2 (0.4%)	
**Histology**	Astrocytoma	194 (37.6%)	-
	Oligoastrocytoma	130 (25.2%)	
	Oligodendroglioma	191 (37.0%)	
	Unknown	1 (0.2%)	

Supplementary Table 6. Twenty-one machine learning algorithms and R package application.

Algorithms/function	R package
boosting	adabag
C50	C50
cforest	party
ctree	party
cvglmnet	glmnet
earth	earth
evtree	evtree
gbm	gbm
glmnet	glmnet
Ibk	Rweka
J48	Rweka
Jrip	Rweka
Kknn	kknn
ksvm	kernlab
lda	MASS
naiveBayes	e1071
OneR	Rweka
PART	Rweka
randomForest	randomForest
randomForestSRC	randomForestSRC
ranger	ranger

Supplementary Table 7. Six candidate algorithms performance in development set for gene expression subtype prediction.

Algorithm	Predicted accuracy	averaged sum of different probabilities per sample
Boosting	68.06%	0.203081
cvglmnet	70.83%	0.198657
cforest	70.83%	0.203108
randomForest	73.61%	0.195134
randomForestSRC	73.61%	0.197521
ranger	73.61%	0.195947
