# Supplementary material for: DNA methylation-based epigenetic signatures predict somatic genomic alterations in gliomas
Source: Nat Commun. 2022 Jul 29;13:4410. doi: 10.1038/s41467-022-31827-x (PMC9338285; doi:10.1038/s41467-022-31827-x)
Supplement: Supplementary file 3 — Description of Additional Supplementary Files [file 41467_2022_31827_MOESM3_ESM.pdf]

Description of Additional Supplementary Files

File Name: Supplementary Data 1
Description: Chromosome enrichment analysis for prediction signatures including binary genomic alterations and gene expression subtypes.

File Name: Supplementary Data 2
Description: GO enrichment analysis results for prediction signatures.

File Name: Supplementary Data 3
Description: Chi-square test between genomic alteration and methyl-based and transc-based gene expression for TCGA-LGG samples.
