# Supplementary material for: DNA methylation-based epigenetic signatures predict somatic genomic alterations in gliomas
Source: Nat Commun. 2022 Jul 29;13:4410. doi: 10.1038/s41467-022-31827-x (PMC9338285; doi:10.1038/s41467-022-31827-x)
Supplement: Supplementary file 7 — Reporting Summary [file 41467_2022_31827_MOESM7_ESM.pdf]

nature portfolio

Corresponding author(s): Erik P. Sulman

Last updated by author(s): Jun 9, 2022

Reporting Summary

Nature Portfolio wishes to improve the reproducibility of the work that we publish. This form provides structure for consistency and transparency in reporting. For further information on Nature Portfolio policies, see our Editorial Policies and the Editorial Policy Checklist.

Statistics

For all statistical analyses, confirm that the following items are present in the figure legend, table legend, main text, or Methods section.

n/a	Confirmed	
☐	☒	The exact sample size (*n*) for each experimental group/condition, given as a discrete number and unit of measurement
☒	☐	A statement on whether measurements were taken from distinct samples or whether the same sample was measured repeatedly
☐	☒	The statistical test(s) used AND whether they are one- or two-sided<br>*Only common tests should be described solely by name; describe more complex techniques in the Methods section.*
☐	☒	A description of all covariates tested
☐	☒	A description of any assumptions or corrections, such as tests of normality and adjustment for multiple comparisons
☐	☒	A full description of the statistical parameters including central tendency (e.g. means) or other basic estimates (e.g. regression coefficient) AND variation (e.g. standard deviation) or associated estimates of uncertainty (e.g. confidence intervals)
☐	☒	For null hypothesis testing, the test statistic (e.g. F, t, r) with confidence intervals, effect sizes, degrees of freedom and P value noted<br>*Give P values as exact values whenever suitable.*
☒	☐	For Bayesian analysis, information on the choice of priors and Markov chain Monte Carlo settings
☒	☐	For hierarchical and complex designs, identification of the appropriate level for tests and full reporting of outcomes
☒	☐	Estimates of effect sizes (e.g. Cohen's *d*, Pearson's *r*), indicating how they were calculated

Our web collection on statistics for biologists contains articles on many of the points above.

Software and code

Policy information about availability of computer code

Data collection	Data were directly downloaded from public data repository. No software was used for data collection.
Data analysis	Data analysis and custom code were applied with R package (version 3.3). R packages utilized include conumee, ChAMP, lumi, wateRmelon, glmnet, Fselector, mlr, adabag, C50, party, earth, evtree, gbm, Rweka, kknn, kernlab, MASS, e1071, randomForest, randomForestSRC, ranger. All R packages are available from CRAN. To facilitate widespread adoption of the UniD platform, we developed an R package for rapid determination of biomarker status in gliomas (available on GitHub, DOI 10.5281/zenodo.6563993).

For manuscripts utilizing custom algorithms or software that are central to the research but not yet described in published literature, software must be made available to editors and reviewers. We strongly encourage code deposition in a community repository (e.g. GitHub). See the Nature Portfolio guidelines for submitting code & software for further information.

Data

Policy information about availability of data

All manuscripts must include a data availability statement. This statement should provide the following information, where applicable:

- Accession codes, unique identifiers, or web links for publicly available datasets
- A description of any restrictions on data availability
- For clinical datasets or third party data, please ensure that the statement adheres to our policy

The data used for training the UniD algorithm include the TCGA glioblastoma data set, which is available in Genomic Data Commons Data Portal with project name as TCGA-GBM (https://portal.gdc.cancer.gov/projects/TCGA-GBM), and the TCGA low grade glioma data set, with project name TCGA-LGG (https://portal.gdc.cancer.gov/projects/TCGA-LGG). This data includes DNA methylation data, copy number variation data, transcriptome profiling data, and clinical information. The processed data are available within the Source Data file. The external validation data set from the NOA04 clinical trial that supports the findings of

this study are available on request from the corresponding author of the paper “NOA-04 randomized phase III trial of sequential radiochemotherapy of anaplastic glioma with procarbazine, lomustine, and vincristine or temozolomide”17. This data includes DNA methylation data, copy number variation data, transcriptome profiling data, and clinical information. The remaining data are available within the Article, Supplementary Information or Source Data file.

Field-specific reporting

Please select the one below that is the best fit for your research. If you are not sure, read the appropriate sections before making your selection.

- [x] Life sciences
- [ ] Behavioural & social sciences
- [ ] Ecological, evolutionary & environmental sciences

For a reference copy of the document with all sections, see nature.com/documents/nr-reporting-summary-flat.pdf

Life sciences study design

All studies must disclose on these points even when the disclosure is negative.

Sample size	No sample-size calculation was performed. All samples from the respective TCGA repositories were used. The number of available samples was validated as sufficient by internal validation of the the UniD model as well as external validation on a different data set, as reported in the manuscript.
Data exclusions	Specific filters were applied to exclude some probes from DNA methylation microarray. The rationale is data read from low quality probes will also have low quality which will impact the study results. Probes not available across different platforms were also excluded to guarantee comparable across platforms.
Replication	All results are computationally derived. Validity is confirmed by replication on a test set and external validation with a data set derived from a different cohort as described above.
Randomization	Samples were stratified sampled into training, development, and test set. The key covariates used to do the stratification including genes mutation status, gene expression subtypes, and copy number variation status.
Blinding	Blinding is not applicable for this study because all data were available before the study. The stratified randomization approach applied during the model building process is enough for study.

Reporting for specific materials, systems and methods

We require information from authors about some types of materials, experimental systems and methods used in many studies. Here, indicate whether each material, system or method listed is relevant to your study. If you are not sure if a list item applies to your research, read the appropriate section before selecting a response.

Materials & experimental systems

n/a	Involved in the study	
x		Antibodies
x		Eukaryotic cell lines
x		Palaeontology and archaeology
x		Animals and other organisms
x		Human research participants
x		Clinical data
x		Dual use research of concern

Methods

n/a	Involved in the study	
x		ChIP-seq
x		Flow cytometry
x		MRI-based neuroimaging
